# Sphingosine 1-Phosphate and Cancer: Lessons from Thyroid Cancer Cells

**DOI:** 10.3390/biom3020303

**Published:** 2013-05-14

**Authors:** Kid Törnquist

**Affiliations:** 1 Department of Biosciences, Åbo Akademi University, Tykistökatu 6A, 20520 Turku, Finland; E-Mail: ktornqvi@abo.fi; Tel./Fax: +35-822-154-748; 2 The Minerva Foundation Institute for Medical Research, Biomedicum Helsinki, 00270 Helsinki, Finland

**Keywords:** Thyroid, cancer, sphingosine 1-phosphate, sphingosine kinase, HERG, follicular, anaplastic

## Abstract

Sphingomyelin is found in the cell membrane of all eukaryotic cells, and was for a long time considered merely as a structural component. However, during the last two decades, metabolites of sphingomyelin, especially sphingosine 1-phosphate (S1P), have proven to be physiologically significant regulators of cell function. Through its five different G protein-coupled receptors, S1P regulates a wide array of cellular processes, ranging from stimulating cellular proliferation and migration, to the inhibition of apoptosis and induction of angiogenesis and modulation of cellular calcium homeostasis. Many of the processes regulated by S1P are important for normal cell physiology, but may also induce severe pathological conditions, especially in malignancies like cancer. Thus, understanding S1P signaling mechanisms has been the aim of a multitude of investigations. Great interest has also been shown in understanding the action of sphingosine kinase (SphK), *i.e*., the kinase phosphorylating sphingosine to S1P, and the interactions between S1P and growth factor signaling. In the present review, we will discuss recent findings regarding the possible importance of S1P and SphK in the etiology of thyroid cancer. Although clinical data is still scarce, our *in vitro* findings suggest that S1P may function as a “double-edged sword”, as the receptor profile of thyroid cancer cells largely determines whether S1P stimulates or blocks cellular migration. We will also discuss the interactions between S1P- and VEGF-evoked signaling, and the importance of a S1P_1_-VEGF receptor 2 complex in thyroid cancer cells.

## 1. Introduction

Sphingomyelin is produced in cells through de novo synthesis in the endoplasmic reticulum (for a recent review see [1]. However, several intermediates in this pathway are important signaling molecules. Sphingomyelin may be hydrolyzed by sphingomyelinases, producing ceramide. This metabolite can further be hydrolyzed to sphingosine. Both ceramide and sphingosine are important regulators of calcium homeostasis, potassium channel activity and apoptosis, to name a few of their cellular effects [2,3,4,5]. Sphingosine can be phosphorylated to sphingosine 1-phosphate (S1P) by sphingosine kinases, of which two isoforms exist. Of these, sphingosine kinase 1 (SphK1) has been investigated more, but novel information regarding the physiological importance of SphK2 is also gathering. The SphKs are predominantly found in the cytosol of resting cells, but upon stimulation, SphK1 is translocated to the plasma membrane and the endoplasmic reticulum [6]. SphK2 has been found in the nucleus and in close proximity to the mitochondria [6], where the produced S1P may regulate histone function and energy metabolism, respectively [7,8].

S1P can be formed in almost all cell types, but erythrocytes and vascular endothelial cells are especially important producers of S1P that is found in the circulation [9,10], where it is bound to albumin and HDL [11]. The concentration of S1P in the circulation markedly exceeds the EC_50_ value for binding to its receptors [12]. Most effects of S1P are due to binding to its receptors on the plasma membrane. Five different, G protein-coupled receptors (S1P_1–5_) have been cloned. These receptors bind to several isoforms of G proteins (G_q/11_, G_i_ or G_s_) and the receptors may have a preference for which G protein they bind. However, the palette of both S1P receptors and G proteins of the cell determines the outcome of the stimulation. Interestingly, the receptors may have diametrically different effects on cell function. This is nicely exemplified by S1P_1_ and S1P_2_: stimulation of S1P_1_ usually potently stimulates migration, whereas stimulation of S1P_2_ has an inhibitory effect on migration. For an extensive review of the different actions of S1P, the reader is referred to several excellent review articles [6,12,13,14,15].

## 2. Sphingosine 1-Phosphate and the Thyroid

The first studies regarding the effect of sphingomyelin derivatives on the thyroid was performed using the well-characterized rat thyroid FRTL-5 cell model. These studies showed that both sphingosine and sphingosylphosphorylcholine evoked a substantial release of sequestered calcium and entry of extracellular calcium [16]. The mechanism of action could not be clarified, but the possibility that either sphingosine or sphingosylphosphorylcholine was converted to a “metabolite” was suggested by the fact that the calcium response was dependent on temperature. Furthermore, a receptor-mediated mechanism was suggested as the calcium response, in part, was sensitive to pertussis toxin.

When S1P became available, we and other researchers unambiguously showed that in FRTL-5 cells, S1P mobilized sequestered calcium from the ER and that this most probably was due to a receptor-mediated mechanism [17,18]. Okajima *et al*. [17] also concluded, that S1P mobilized calcium through an inositol 1,4,5-trisphosphate (IP_3_)-mediated mechanism in these cells. We could not confirm these observations, possibly due to methodological differences. In rat thyroid PCCl3 cells, S1P does slightly increase IP_3_ formation [19]. However, other reports suggested that S1P mobilized calcium through an IP_3_-independent mechanism [20], and we could show that intracellular S1P might be involved in releasing intracellular calcium from the FRTL-5 cells [21]. The effect of intracellular S1P on calcium release is still enigmatic, as microinjections of S1P do induce calcium release in HEK-293 cells, although an intracellular calcium-mobilizing receptor for S1P is yet to be found [22].

In addition to mobilizing sequestered calcium, exogenous S1P has been shown to stimulate an increase in the expression of c-fos and DNA synthesis in FRTL-5 cells [18], and also to activate Na^+^-H^+^ exchange [23], an important step in activation of proliferation. Furthermore, S1P stimulates the production of hydrogen peroxide by a calcium-dependent mechanism [17]. Interestingly, Kimura *et al*. showed that stimulating the cells with TNFα inhibited TSH-evoked hydrogen peroxide production in FRTL-5 cells [24]. The effect of TNFα was mimicked by ceramide. As the production of hydrogen peroxide in these cells was dependent on calcium [25], it is possible that TNFα attenuated hydrogen peroxide production by decreasing calcium entry in the cells. We base this suggestion on the fact that our results have shown that TNFα and ceramide potently hampers calcium entry in FRTL-5 cells, probably by blocking a potassium channel and depolarizing the membrane potential, thus decreasing the electrochemical gradient for calcium entry [26].

The receptor profile of FRTL-5 and PCCl3 thyroid cells is identical: both cell lines express only S1P_2_ and S1P_3_. As these receptors activate several different G proteins, including G_i_ [27], they potently inhibit the TSH-evoked activation of adenylate cyclase and cAMP production [17,18,19]. Taken together, the results obtained with rat thyroid cells clearly show that S1P can modulate thyroid function. However, it is important to note that very little is known in regard to the effect of S1P on normal human thyroid cells, except that these cells express all S1P receptors, albeit S1P_4_ at very low levels, and that S1P induces calcium responses also in primary cultures of normal thyroid cells [28].

## 3. Sphingosine 1-Phosphate and Thyroid Cancer

The importance of S1P in regulating proliferation, invasion and migration in different types of cancer cells has been the subject of a multitude of investigations. The S1P pathway has been observed to be deregulated in several forms of cancers, including breast, ovary, and different forms of cancer in the gastrointestinal tract. This deregulation occurs at different parts of the pathway and might be due to overexpression of SphK1, deregulated S1P metabolizing enzymes, or mutations or changes in the expression of S1P receptors. For extensive reviews, please see [14,15,29]. In regard to thyroid cancer, only one report on the importance of S1P is available. In a recent report by Guang *et al*., the expression of SphK1 was shown to be upregulated in thyroid cancer and to correlate with malignancy. Furthermore, the expression of SphK1 correlated significantly with the expression of proliferating cell nuclear antigen (PCNA), indicating that proliferation of thyroid cancer cells is associated with the expression of SphK1 [30]. We have made a preliminary analysis of S1P receptor expression in a small sample of thyroid tumors, but were unable to detect any significant changes in the expression of S1P receptors in cancerous thyroid tissues (Balthasar and Törnquist, unpublished observations).

### 3.1. Receptor Profile in Thyroid Cancer Cells

In all the human thyroid cancer samples we analyzed, all five S1P receptors were expressed. As the expression was analyzed using qPCR from tissue samples, we cannot exclude that our results are in part due to the existence of receptors also on other cell types than thyroid epithelial cells (Balthasar and Törnquist, unpublished observations). However, to obtain a more reliable picture of receptor expression, we analyzed several thyroid cancer cell lines and human thyroid cells in primary culture. In human primary cultures of thyroid cells, all receptors were expressed, albeit the expression of S1P_4_ was minimal. Furthermore, most cell lines investigated (including, papillary, follicular and anaplastic thyroid cancer cell lines) expressed abundantly S1P_1–3_, the receptors that potently modulate migration of cells [28,31].

### 3.2. Effects on Proliferation and Migration

Several studies have shown, that administration of exogenous S1P may enhance proliferation of both normal and cancer cells [14,27]. In normal human thyroid cells in primary culture and in the normal thyroid Nthy-ori 3-1 cell line, administration of exogenous S1P was without any effects on proliferation [28]; Asghar and Törnquist, unpublished observations). In follicular ML-1 cells, anaplastic FRO and WRO thyroid cancer cells, and in papillary NPA cancer cells, S1P slightly attenuated proliferation [28]. However, as the origin of all but the ML-1 cells was dubious, we investigated some other original thyroid cancer cells lines. In follicular FTC-133 cells, and anaplastic C643 and THJ-16T thyroid cancer cells, S1P was without an effect on proliferation [31]. Thus, although the number of cell lines tested is limited, the effect of S1P on the proliferation of thyroid cancer cells seems minimal. 

If S1P had, at most, a very modest effect on proliferation, the effect on migration was much more prominent. In almost all cell lines tested, S1P potently attenuated migration. The inhibitory effect was investigated in detail using anaplastic thyroid cancer C643 cells. In these cells, the inhibitory effect was crucially dependent on the expression of S1P_2_ and on Rho activity [31], as has been shown also for other types of cancer cells [32]. In addition, S1P inhibited Rac activity [31].

In the follicular thyroid ML-1cancer cell line, on the other hand, administration of exogenous S1P potently stimulated migration. The migratory response was mediated by S1P_1_ and a pertussis toxin-dependent mechanism. Downstream from the receptor, Rac, PKCα and ERK1/2 were important for S1P-evoked migration, as well as the activation of PI3K and Akt [28,33]. The importance of the PKC-activated pathway was underlined by results showing that direct activation of PKC with the diacylglycerol analogue 1-oleyl-2-acetyl-sn-glycerol (OAG) potently stimulated migration. However, in follicular thyroid FTC-133 cancer cells, which have a receptor profile very similar to the ML-1 cells, S1P potently attenuated migration. Furthermore, the receptor profile in the anaplastic C643 thyroid cancer cell line (in which S1P also inhibited migration) was similar to that in ML-1 cells. This is, in our opinion, a very important and disappointing observation, as it suggests that the S1P receptor profile *per se* probably cannot be used as a marker for a migratory phenotype of thyroid cancer cells. 

### 3.3. Importance of Sphingosine Kinase

Several studies have suggested that SphK1 may have an oncogenic potential or even be classified as an oncogene (although no mutated forms of SphK have so far been reported). By measuring tumor growth in immunodeficient mice and colony formation in soft agar, it was concluded that SphK might be an oncogene [34]. Furthermore, overexpression of SphK in NIH3T3 cells revealed an enhanced cell cycle transition [35], and expression of SphK was considered a marker of poor prognosis in breast cancer [36], and correlated with malignancy in thyroid cancer [30]. The effect of overexpression of SphK and enhanced production of S1P most probably affected cancer cells by an autocrine effect of S1P (see [37]).

The study by Guan *et al*. [30] clearly indicated that silencing of SphK1 attenuated the proliferation of several follicular and anaplastic thyroid cancer cell lines. In clinical samples, overexpression of SphK1 correlated significantly with the expression of PCNA, suggesting a strong association between SphK1 overexpression and proliferation of thyroid cancer cells. Furthermore, using thyroid cell lines Guan *et al*. also observed a decreased β-catenin-TCF/LEF-induced transcriptional activity in SphK1 knock-down cells, resulting in decreased expression of cyclin D-1 and c-myc. Furthermore, they also observed decreased Akt phosphorylation, and dephosphorylation and activation of GSK-3 [30].

In our studies, overexpression of SphK1 in follicular ML-1 and FTC-133 thyroid cancer cells resulted in a decreased proliferation, compared with mock-transduced cells or cells transduced with the inactive G82D mutant of SphK1 [33]. The reason for the difference from the results obtained by Guan *et al*. is presently not known. One possibility is a different set-up of S1P receptors in the cell lines used in the two studies. Another possibility is that cross talk between S1P receptors and some growth factor receptors (see [38]) resulted in an enhanced proliferative potential in the cells used by Guan *et al*. [30]. However, the migration of ML-1 cells overexpressing SphK1 was significantly increased. Further investigations showed that this effect was due to autocrine S1P signaling. The migration was attenuated by pretreatment with pertussis toxin, by pharmacologically blocking S1P_1_ and ERK1/2, by siRNA against PKCα, and finally by blocking the ATP-binding cassette transporter C1 (ABCC1) [33]. Other investigations have shown that ABCC1, and the ATP-binding cassette transporter A1 (ABCA1), both are involved in transporting S1P out of cells, resulting in autocrine S1P signaling [39,40]. Taken together, the results obtained by us and by Guan *et al*. [30] suggest, that overexpression of SphK and autocrine S1P signaling may be detrimental in the etiology of thyroid cancer.

As is always the case in biology, nothing is as straightforward as it first seems. In the study by Bergelin *et al*. [33], overexpression of SphK enhanced migration. However, in a study by Asghar *et al*. [31], pharmacological inhibition of SphK1, on the other hand, enhanced migration of the anaplastic C643 thyroid cancer cells, probably due to decreased autocrine S1P signaling. Addition of exogenous S1P to these cells resulted in a decreased migration. Furthermore, our results showed that in these cells, S1P predominantly activated S1P_2_. Our results thus indicate that activation of SphK1 may, in some cell types, evoke an anti-migratory effect. We conclude that the receptor profile of the cells, again, may decide the outcome of such an autocrine S1P signaling. Thus, it probably is advisable to proceed with caution before considering the use of SphK-inhibitors in the treatment of thyroid cancer. 

### 3.4. Signaling cross Talk with VEGF

Signaling between G protein-coupled receptors and tyrosine kinase receptors probably is a common mechanism regulating cell fate (see review by Pyne *et al*. [41]). In an early article, Berk’s group showed cross talk between S1P signaling and EGF on Erk1/2 signaling in bovine aortic endothelial cells [42]. Furthermore, cross-communication between S1P-receptors and both platelet-derived growth factor (PDGF)-, transforming growth factorβ (TGFβ), and insulin-like growth factor receptors have also been shown [43,44,45], suggesting that also the receptors for S1P may participate in cross communication with tyrosine kinase receptors. For further details, the readers are referred to reviews by Lebman and Spiegel [46], and Pyne and Pyne [38]. 

Signaling between S1P receptors and growth factor receptors can be either sequential or integrative [46,47]. Sequential signaling means that a growth factor binds to and activates its receptor, resulting in the activation of SphK. The produced S1P is then transported out from the cell and activates its own receptor through an autocrine or paracrine mechanism. Integrative signaling, on the other hand, is the result of activation of a complex containing both a growth factor receptor and a S1P receptor. The activation of the complex is most probably bidirectional, meaning that activation of either one receptor will result in activation of the other, and that activation of both receptors is necessary for the activation of downstream signaling pathways [46,47].

Several investigations have shown that thyroid carcinoma cells express receptors for VEGF and that the cells also express and secret VEGF [48,49,50]. Furthermore, S1P and VEGF have been shown to cooperate to regulate cellular functions in several cell types, both normal cells and in malignant cell types. In, e.g., endothelial cells, S1P transactivates and phosphorylates VEGF receptor 2 [42,51]. VEGF, in turn, regulates the expression of S1P_1_ and S1P_3_ [52,53,54]. In addition, VEGF may enhance SphK activity and the production of S1P [55].

We thus wanted to investigate whether S1P receptors and receptors for VEGF interacted in ML-1 thyroid cancer cells. In the first study, we showed that ML-1 cells express VEGF receptor 2 (VEGFR2) and constitutively secrete VEGF [56]. Furthermore, S1P stimulated a small, but significant increase in the secretion of VEGF-A. VEGFR2 activity was also of crucial importance for migration, as inhibition of VEGFR2 pharmacologically, or sequestering VEGF with an antibody, clearly decreased both basal and S1P evoked migration. Interestingly, the expression of S1P receptors seemed to be, at least in part, regulated by VEGFR2 activity, as blocking VEGFR2 rapidly decreased the expression of S1P_1_ but increased the expression of S1P_3_ [56]. Adding to the complexity was our observation that stimulation of ML-1 cells with S1P, in turn, transiently increased VEGFR2 expression through a mechanism dependent on S1P_3_, PKCα and ERK1/2 [57].

The above results suggested that S1P receptors and VEGFR2 might function as a complex in regulating ML-1 cell migration. This is strengthened by our observation that S1P, indeed, phosphorylated VEGFR2, and that S1P_1_ and VEGFR2 colocalized at the plasma membrane, as shown by immunocytochemistry [57]. In addition, several other investigations have shown an intimate relationship between S1P receptors and growth factor receptors, e.g., the PDGFβ-receptor, the EGF-receptor and the IGF receptor this is transactivation through SK [44,58,59].

To further investigate the interactions between VEGFR2 and S1P_1_, we immunoprecipitated VEGFR2 and showed that S1P_1_ also was immunoprecipitated. In addition, both ERK1/2 and PKCα was coimmunoprecipitated in the complex [57]. Immunoprecipitation of VEGFR2 also resulted in detectable amounts of S1P _2,3_,_5_ in the complex. When S1P_1_ was immunoprecipitated, VEGFR2, PKCα and ERK1/2 was coimmunoprecipitated. Thus, at least in ML-1 cells, VEGFR2 and S1P_1_ are in a complex together with PKCα and ERK1/2, *i.e*., the signaling molecules important for ML-1 cell migration. 

**Figure 1:**
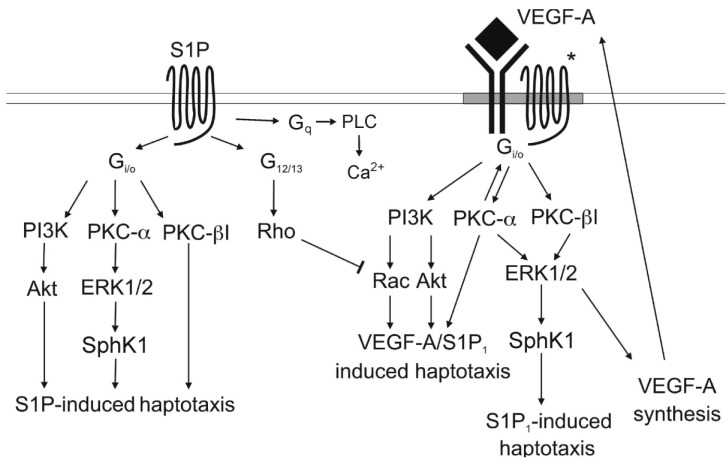
Schematic presentation of the different signaling pathways employed by S1P and VEGF in stimulating migration of follicular ML-1 thyroid cancer cells. The * in the Figure denotes a complex between S1P receptors and VEGF receptor 2.

We next investigated by which mechanisms VEGFR2 and S1P_1_ interacted to regulate ML-1 cell migration. Our investigations showed that treatment of the cells with pertussis toxin (Ptx) or a PKCα/β inhibitor, inhibited VEFG-A-evoked ERK1/2 phosphorylation in a manner similar to that of S1P. Previous investigations have also showed that both IGF and PDGFβ may phosphorylate ERK1/2 by a Ptx-dependent mechanism [44,58]. Furthermore, downregulation of PKCα or PKCβ attenuated both the SEW-2871 (a S1P_1_ agonist) and VEGF-A-evoked ERK1/2 phosphorylation. Furthermore, both S1P and VEGF-A-evoked haptotaxis was attenuated by Ptx [57]. A schematic presentation of the S1P- and VEGF-evoked signaling in regard to thyroid cancer ML-1 cell migration is presented in Figure 1.

Thus, S1P1-VEGFR2 cross talk seems to be integrative and bidirectional: both S1P- and SEW-induced ERK1/2 phosphorylation and haptotaxis was inhibited by a VEGFR2 inhibitor, whereas VEGF-A-evoked ERK1/2 phosphorylation and haptotaxis was inhibited by S1P_1_ siRNA. As thyroid cancer cells also may express receptors for other growth factors, an interesting question is if and how S1P interacts with these receptors, and how such an interaction could modulate either proliferation or migration of thyroid cancer cells. 

## 4. Cross Talk with Ion Channels: HERG

HERG potassium channels have been implicated to participate in the regulation of migration and proliferation of several types of cancer [60,61]. Furthermore, the enhanced expression of HERG has been shown to correlate with a worse prognosis in, e.g., *glioblastoma multiforme* [62]. As the possible significance of HERG in thyroid cancer has not been evaluated, we investigated this in anaplastic cancer cells. Our data showed that both normal human thyroid cells, as well as thyroid cancer cells, express HERG channels [31]. Interestingly, HERG-like currents did not parallel the channel expression. However, in both anaplastic and follicular cancer cells, inhibition of the HERG channels with E-4031 resulted in a decreased migration of the cells. Interestingly, incubation of the anaplastic C643 thyroid cancer cells with S1P resulted in a transient decrease in the expression of the HERG protein. A similar S1P-evoked reduction in HERG protein expression was seen in HEK cells overexpressing HERG, and in these cells, S1P also decreased migration. The reason for the downregulation is not clear, but could be due to S1P-evoked receptor activation, the activation of phospholipase C and the production of diacylglycerol, resulting in internalization and degradation of HERG channels (see [63]). Whether the link between S1P-receptor signaling and HERG internalization can be of clinical importance is an open question. Another interesting observation is that HERG channel activity may enhance VEGF secretion [62]. As VEGF may activate VEGFR2 through an autocrine mechanism in thyroid cancer cells, enhanced HERG expression or activity could then worsen the prognosis of the disease. This observation may possibly be of clinical significance.

## 5. Concluding Remarks

The studies described above clearly suggest that SphK1 and S1P may be important in the etiology of thyroid cancer, and in the regulation of both invasion and migration of thyroid cancer cells. However, the fact that migration of thyroid cancer cells of different cancer forms, but with very similar S1P receptor profiles, may either be inhibited or stimulated by S1P, is a matter of concern. This suggests that the receptor profile *per se* cannot be used as a marker for discerning a more migratory phenotype of cancer cells. Similarly, the observations that overexpression of SphK1 also may either have an inhibitory or stimulatory effect on migration, might be problematic if inhibition of SphK is to be used in clinical settings: the treatment might, in fact, enhance instead of inhibit migration and metastasis of cancer cells. However, the intimate cross-communication between S1P_1_ and VEGFR2 might prove to be an advantage in the search for an effective treatment for thyroid cancer. Clearly, more investigations are needed to clarify if inhibition of SphK1 or S1P-receptors will be of clinical significance in the treatment of thyroid cancer. 
